# Risk of physical health comorbidities in autistic adults: clinical nested cross-sectional study

**DOI:** 10.1192/bjo.2024.777

**Published:** 2024-10-23

**Authors:** Megan Hunt, Jack F. G. Underwood, Leon Hubbard, Jeremy Hall

**Affiliations:** Foundation Programme, North Bristol NHS Trust, UK; and Neuroscience & Mental Health Innovation Institute, Division for Psychological Medicine and Clinical Neuroscience, Cardiff University, UK; Neuroscience & Mental Health Innovation Institute, Division for Psychological Medicine and Clinical Neuroscience, Cardiff University, UK; National Centre for Mental Health, Division for Psychological Medicine and Clinical Neuroscience, Cardiff University, UK

**Keywords:** Autism, physical health, co-occurring conditions

## Abstract

**Background:**

Physical health conditions are more common in individuals with autism. Some, like epilepsy, have considerable evidence supporting their increased prevalence, but many diseases lack literature to make strong conclusions.

**Aims:**

To investigate the prevalence of physical health comorbidities in autism.

**Method:**

We undertook a nested cross-sectional study, using a sample from the National Centre for Mental Health database. It included participants from England and Wales who reported a clinician-made diagnosis of autism (*n* = 813), and a control sample without autism or mental illness (*n* = 2781). Participants had provided a medical history at enrolment. Analysis was carried out by binomial logistic regressions controlling for age, gender, smoking status, and antipsychotic and mood stabiliser use. A subanalysis of individuals with concurrent intellectual disability (*n* = 86) used binomial logistic regression with the same control variables.

**Results:**

Many physical health conditions were significantly more common in autism. Sixteen out of 28 conditions showed increased odds, with the highest odds ratios observed for liver disease, chronic obstructive pulmonary disease, kidney disease, osteoporosis and rheumatoid arthritis. A subanalysis demonstrated a similar pattern of physical health in individuals with autism with and without concurrent intellectual disability. Some conditions, including osteoporosis, hyperthyroidism, head injury and liver disease, had larger odds ratios in individuals with concurrent intellectual disability.

**Conclusions:**

Physical health conditions occur more commonly in individuals with autism, and certain conditions are further increased in those with concurrent intellectual disability. Our findings contribute to prior evidence, including novel associations, and suggest that people with autism are at greater risk of physical health problems throughout adulthood.

Autism spectrum disorder (hereafter ‘autism’ or ‘autistic’, reflecting reported community preference for identity-first language^1^) is a neurodevelopmental condition that is characterised by behavioural and social communication features. It is a common condition, with a reported prevalence of approximately 1%,^2^ and has a male:female ratio of about 3:1.^3^ There is much research into psychiatric comorbidities in autism, showing a high burden of co-occurring mental health conditions;^4^ however, research into physical health conditions is more limited.

A recent umbrella systematic review suggests that there is an increased prevalence of physical conditions in autism, but this paper also serves to highlight vast gaps in the literature, with no adequate systematic reviews on many major conditions, such as heart disease.^5^ Many large database studies similarly allow us to conclude higher rates of physical health conditions in the autistic community, finding increased prevalence of conditions such as gastrointestinal disorders, neurological disorders (e.g. epilepsy), diabetes, thyroid disorders and metabolic disorders (e.g. dyslipidaemia);^6–12^ however, we are limited in our ability to make conclusions about specific health conditions, as many are not well studied. Epilepsy is the most well-researched physical condition in autism, with multiple meta-analyses and individual studies documenting increased odds compared with controls or the general population (often with large odds ratios),^6–8,11–14^ and findings suggest that those who are female or have concurrent intellectual disability are at an even greater risk.^15,16^

Considering gastrointestinal disorders, one meta-analysis found increased odds of constipation, diarrhoea and abdominal pain in children with autism;^17^ another found that adults with autism are more likely to have many conditions, such as irritable bowel syndrome, hernias, gallbladder disease and chronic constipation;^10^ and multiple studies suggest inflammatory bowel disease to be more common in autism.^6,18,19^ Several studies find increased risk of hyperlipidaemia/dyslipidaemia,^7,11,12,14,20^ hypertension^7,8,20^ and various heart diseases^7–9,21^ in individuals with autism, suggesting that these could be more common, but the lack of studies and the existence of several studies with contradictory findings^9,11,14,22^ preclude strong conclusions. Autoimmune diseases seem to be more common in individuals with autism,^7,23^ and autism is more common in those with autoimmune diseases,^24^ although it is unclear from research if this applies to a select few or to all autoimmune diseases.

For several conditions, much of the existing evidence is conflicting: asthma has multiple studies supporting its increased prevalence in autism,^9,21,25^ but two recent meta-analyses conclude no association between autism and asthma.^26,27^ A recent review of diabetes concluded uncertainty over whether it is more common in autism^28^ and cancer shows a similarly mixed picture, with some studies showing increased^8,29^ and some showing decreased^7,12^ rates of cancer in individuals with autism. Most notably, conditions of old age are rarely investigated, and although one study in people aged >65 years found increased odds of a range of age-related conditions like osteoporosis, osteoarthritis, Parkinson's disease, chronic obstructive pulmonary disease (COPD) and cognitive disorders,^8^ and another in people aged >45 years found increased hazard ratios for conditions including heart failure, cerebrovascular disease and COPD,^9^ few of these results are replicated elsewhere.

## Aims

For many co-occurring health conditions in autism, gaps in the literature prevent conclusions or interpretation of underlying mechanisms. This study therefore aims to compare the physical health of individuals with and without autism, using a nested cross-sectional design on a large, prospectively recruited e-cohort of individuals across a broad range of ages, to assess whether specific conditions or clusters of conditions are more common in individuals with autism.

## Method

### Study population and measures

The sample was drawn from the National Centre for Mental Health (NCMH) database. The NCMH is a Welsh Government-funded research centre investigating neurodevelopmental, adult psychiatric and neurodegenerative psychiatric disorders across the lifespan.^30^ The cohort in the database consists of individuals aged 4 years or older, who have experienced or are related to someone who has experienced one of these disorders, as well as volunteer control participants who have not experienced any disorder.^30^ Participants were recruited systematically to the NCMH through disease registers, clinical note screening and identification by clinical care teams, and non-systematically through media advertisements, posters/leaflets in National Health Service (NHS) waiting rooms, voluntary organisations and contacting of individuals involved in previous studies within the Division of Psychological Medicine and Clinical Neurosciences, Cardiff University, UK.^30^ All individuals in this study provided written informed consent after viewing the patient information sheet or, for those under 16 years of age or lacking sufficient mental capacity, assent was provided where possible, and written consent was obtained from a nominated individual, such as the next of kin, a family member or a carer.^30^ Participants underwent a standardised interview establishing personal and family history of mental illness and medication profile, and were also given a standardised self-report questionnaire to complete.^30^

The NCMH received a favourable ethical opinion from the Wales Research Ethics Committee 2 on 25 November 2016 (approval number 16/WA/0323). This project utilised data held within the NCMH ethical approval, as part of existing questionnaires. An application for data access was made in December 2021, and approved after internal board review in January 2022. The NCMH database was interrogated and individuals were extracted for inclusion in this study if they had reported having a clinical diagnosis of autism and had filled in any of the medical history section of the questionnaire (*n* = 813) (full recruitment method provided in the Supplementary Material, available at https://doi.org/10.1192/bjo.2024.777). An unmatched control sample of individuals without autism and with no mental health or neurodevelopmental conditions (*n* = 2781) was also obtained from the NCMH database. Any individuals who were completely missing health data were excluded from analysis. Where individuals had only completed part of the questionnaire, they were included in analysis for physical conditions where they had data available.

Following consent, trained researchers administered a standardised interview assessment to gather data on the participant's diagnostic history at enrolment to NCMH. The NCMH Brief Assessment captures information on lifetime physical health conditions in its medical history section at enrolment to NCMH, and this was used to derive the outcome measure of physical health conditions in the sample. Participants are given a list of conditions and told to indicate whether they have ever been told by a doctor or health professional that they have the condition, allowing for a binary outcome of either presence or absence of the condition. Questions on lifetime diagnosis are asked as ‘Has a doctor or health professional ever given you a diagnosis of [listing a broad range of psychiatric and physical health conditions, one at a time, including autism]?’ Further information regarding diagnosis, symptoms, treatment and outcomes was obtained from clinical records, where appropriate consent had been obtained to do so. After assessment of the data (see Supplementary Material), 28 physical health variables were included for statistical analysis.

### Statistical analysis

Initially, data were plotted to visualise and compare group frequencies, followed by *χ*^2^-test to assess the association between autism and physical health, with a significance level of 5%. Binomial logistic regression was used to calculate odds ratios for each condition in autism, with a 95% confidence interval. Two logistic regression models were used. The first model looked at the odds of each condition in autism and included age and gender as covariates in the model. Autism diagnosis was the independent variable and physical health diagnoses were the dependent variable. A second multivariable logistic regression model further included smoking status, mood stabiliser use and antipsychotic use, as well as age and gender as covariates, again with autism diagnosis as the independent variable and physical health diagnoses the dependent variable. These covariates were selected as known risk factors for a wide range of diseases (see Supplementary Material for explanation and citations). Although some mood stabilisers are also anticonvulsants, removing mood stabilisers as a control variable when investigating epilepsy had little effect on the odds ratio in a *post hoc* assessment (see Supplementary Material), so it was deemed most appropriate to continue using the same model for all conditions.

### Intellectual disability subanalysis

Additional subanalyses were conducted to investigate the effects of intellectual disability. Binomial logistic regression models were run in which each physical health condition outcome (dependent variable) was assessed, comparing controls (*n* = 2781) with individuals with autism with (*n* = 86) and without (*n* = 727) intellectual disability, controlling for age, gender, smoking status and antipsychotic and mood stabiliser use. A direct comparison model between autism with intellectual disability and isolated autism was not utilised because of concerns around collider biases between autism and intellectual disability. In the subanalysis, a further five conditions were identified as having no cases among individuals with concurrent intellectual disability, and therefore were not modelled.

Benjamini–Hochberg correction was carried out to adjust for multiple hypothesis testing and reduce the chance of a type 1 error, with an initial *α* of 0.05. In the main analysis, 75 tests were run (25 tests per model) and included in the correction, leading to a false discovery rate correction of 0.029. In the subanalysis, 20 tests were run, leading to a false discovery rate correction of 0.029. Data were analysed with IBM SPSS Statistics Version 27 for Windows.^31^

## Results

Data were collected for 3674 participants. After deletion of duplicates and individuals with no recorded physical health data, a total sample of 3594 individuals remained. A total of 813 participants met the inclusion criteria for the autism group, and these were compared against 2781 controls.

The mean age in the autism sample was 33.73 years (range 11–97 years, s.d. 13.45). The mean age in the control sample was 49.61 years (range 6–93 years, s.d. 18.74). The autism group was 41.9% female, 53.3% male and 4.4% gender variant/non-conforming/transgender male/transgender female; the control group was 65.8% female, 33.0% male and 0.9% gender variant/non-conforming/transgender male/transgender female. The male:female ratio in the autism group was 1.27:1.

On initial analysis with *χ*^2^-test (Table 1), the autism sample was noted to have a significantly greater prevalence (*P* < 0.001) of asthma (33.2 *v.* 16.3%), epilepsy (8.0 *v.* 1.8%), head injury (16.4 *v.* 3.2%), migraine headaches (33.2 *v.* 15.5%), inflammatory bowel disease (7.2 *v.* 3.1%), liver disease (1.9 *v.* 0.4%) and other autoimmune conditions (5.2 *v.* 2.2%) than the control sample. It was also observed that the control group had a significantly higher prevalence of cancer (6.3 *v.* 1.4%), heart disease (4.0 *v.* 1.5%), hypertension (16.7 *v.* 12.0%) and osteoarthritis (8.9 *v.* 5.5%) than the autism group. All other individual conditions showed no significant difference between the autism and control groups.

**Table 1:** Frequency of physical health conditions in cases and controls, and results of the *χ*^2^ analysis

Physical health condition	Frequency, *n* (%)		Pearson *χ*^2^ value	*P*-value
	Autism	Controls		
Asthma	264 (33.2)	453 (16.3)	108.902	<0.001*
Breast cancer	0 (0.0)	56 (2.0)		
Cancer (other)	11 (1.4)	175 (6.3)	30.741	<0.001*
COPD	4 (1.0)	29 (1.2)	0.178	0.673
Diabetes type 1	7 (0.9)	20 (0.7)	0.199	0.656
Diabetes type 2	35 (4.4)	128 (4.6)	0.082	0.775
Elevated lipids/cholesterol	72 (9.0)	252 (9.1)	0.003	0.959
Epilepsy/seizure disorder	63 (8.0)	50 (1.8)	76.301	<0.001*
Gastric/duodenal ulcers	28 (3.5)	68 (2.4)	2.833	0.092
Head injury	68 (16.4)	79 (3.2)	125.765	<0.001*
Heart disease	12 (1.5)	110 (4.0)	11.290	<0.001*
Hypertension	95 (12.0)	464 (16.7)	10.595	0.001*
Kidney disease	13 (1.6)	36 (1.3)	0.508	0.476
Liver disease	15 (1.9)	12 (0.4)	17.369	<0.001*
Memory loss (dementia)	10 (1.3)	17 (0.6)	3.511	0.061
Migraine headaches	262 (33.2)	428 (15.5)	123.641	<0.001*
Meningitis	6 (1.4)	21 (0.9)	1.220	0.269
Osteoarthritis	44 (5.5)	247 (8.9)	9.371	0.002*
Osteoporosis	14 (1.8)	54 (1.9)	0.127	0.722
Rheumatoid arthritis	26 (3.3)	73 (2.6)	0.981	0.322
Stroke/haemorrhage	11 (1.4)	39 (1.4)	0.002	0.968
Overactive thyroid/hyperthyroid	6 (0.8)	46 (1.7)	3.458	0.063
Underactive thyroid/hypothyroid	40 (5.0)	147 (5.3)	0.095	0.758
Inflammatory bowel disease	30 (7.2)	75 (3.1)	17.097	<0.001*
Other autoimmune condition	22 (5.2)	54 (2.2)	12.553	<0.001*

Human immunodeficiency virus, Parkinson's disease and multiple sclerosis are not included because of absolute frequency counts <5 allowing back-identification, and were not significantly different. COPD, chronic obstructive pulmonary disease.

* Indicates *P*-values that are significant after Benjamini–Hochberg correction (α 0.029).

The initial logistic regression model controlling for age and gender found increased odds of 17 of the 28 physical health conditions in the autism group (Table 2). The largest odds ratios were observed for liver disease (odds ratio 11.55, 95% CI 4.36–30.60, *P* < 0.001), head injury (odds ratio 5.03, 95% CI 3.30–7.67, *P* < 0.001), osteoporosis (odds ratio 5.16, 95% CI 2.37–11.25, *P* < 0.001), kidney disease (odds ratio 4.97, 95% CI 2.13–11.59, *P* < 0.001) and memory loss/dementia (odds ratio 4.93, 95% CI 1.80–13.51, *P* = 0.002). All 17 conditions remained significant after Benjamini–Hochberg correction.

**Table 2:** Odds ratios and *P*-values from binomial logistic regression

Physical health condition	Model 1		Model 2	
	Adjusted odds ratio (95% CI)	*P*-value	Adjusted odds ratio (95% CI)	*P*-value
Asthma	2.36 (1.90–2.92)	<0.001*	2.36 (1.84–3.02)	<0.001*
Cancer (other)	0.71 (0.34–1.47)	0.357	0.54 (0.20–1.43)	0.215
COPD	3.31 (0.88–12.48)	0.077	7.42 (1.79–30.77)	0.006*
Diabetes type 1	1.22 (0.43–3.41)	0.710	0.64 (0.14–3.00)	0.573
Diabetes type 2	2.74 (1.67–4.48)	<0.001*	2.32 (1.28–4.21)	0.005*
Elevated lipids/cholesterol	3.89 (2.68–5.66)	<0.001*	2.75 (1.74–4.34)	<0.001*
Epilepsy/seizure disorder	3.84 (2.35–6.29)	<0.001*	3.44 (1.95–6.04)	<0.001*
Gastric/duodenal ulcers	2.65 (1.47–4.77)	0.001*	2.37 (1.18–4.76)	0.016*
Head injury	5.03 (3.30–7.67)	<0.001*	3.84 (2.30–6.39)	<0.001*
Heart disease	0.91 (0.38–2.13)	0.818	0.57 (0.17–1.94)	0.367
Hypertension	2.38 (1.72–3.28)	<0.001*	2.11 (1.44–3.09)	<0.001*
Kidney disease	4.97 (2.13–11.59)	<0.001*	4.96 (1.91–12.85)	<0.001*
Liver disease	11.55 (4.36–30.60)	<0.001*	10.96 (3.72–32.25)	<0.001*
Memory loss (dementia)	4.93 (1.80–13.51)	0.002*	3.71 (1.13–12.22)	0.031
Migraine headaches	3.66 (2.92–4.59)	<0.001*	3.45 (2.66–4.48)	<0.001*
Meningitis	0.85 (0.26–2.79)	0.783	1.06 (0.28–4.01)	0.928
Multiple sclerosis	0.70 (0.08–6.07)	0.743	0.00 (0.00)	0.991
Osteoarthritis	2.84 (1.81–4.43)	<0.001*	2.59 (1.50–4.46)	<0.001*
Osteoporosis	5.16 (2.37–11.25)	<0.001*	4.66 (1.76–12.33)	0.002*
Rheumatoid arthritis	3.49 (1.88–6.47)	<0.001*	4.55 (2.33–8.88)	<0.001*
Stroke/haemorrhage	1.93 (0.82–4.56)	0.134	1.22 (0.39–3.83)	0.730
Overactive thyroid/hyperthyroid	1.93 (0.74–5.02)	0.177	2.11 (0.68–6.55)	0.196
Underactive thyroid/hypothyroid	2.70 (1.75–4.17)	<0.001*	2.18 (1.28–3.70)	0.004*
Inflammatory bowel disease	2.97 (1.74–5.06)	<0.001*	2.10 (1.06–4.17)	0.035
Other autoimmune condition	3.79 (2.00–7.17)	<0.001*	3.38 (1.60–7.15)	0.001*

Model 1 controls for age and gender; model 2 controls for age, gender, antipsychotic use, mood stabiliser use and smoking status. COPD, chronic obstructive pulmonary disease.

* Indicates *P*-values that are significant after Benjamini–Hochberg correction (α 0.029).

The medication- and smoking-adjusted logistic regression model, which controlled for age, gender, smoking, antipsychotic use and mood stabiliser use, found autism increased the odds for 18 out of 28 physical health conditions (the same conditions as the first model, with the addition of COPD). Only 16 remained significant after Benjamini–Hochberg correction (Table 2). In this model, the greatest increase in odds ratios associated with autism were observed for liver disease (odds ratio 10.96, 95% CI 3.72–32.25, *P* < 0.001), COPD (odds ratio 7.42, 95% CI 1.79–30.77, *P* = 0.006), kidney disease (odds ratio 4.96, 95% CI 1.91–12.85, *P* < 0.001), osteoporosis (odds ratio 4.66, 95% CI 1.76–12.33, *P* = 0.002) and rheumatoid arthritis (odds ratio 4.55, 95% CI 2.33–8.88, *P* < 0.001). Neither regression model showed any condition to be significantly lower odds in the autism group.

### Intellectual disability subanalysis

**Fig. 1:**
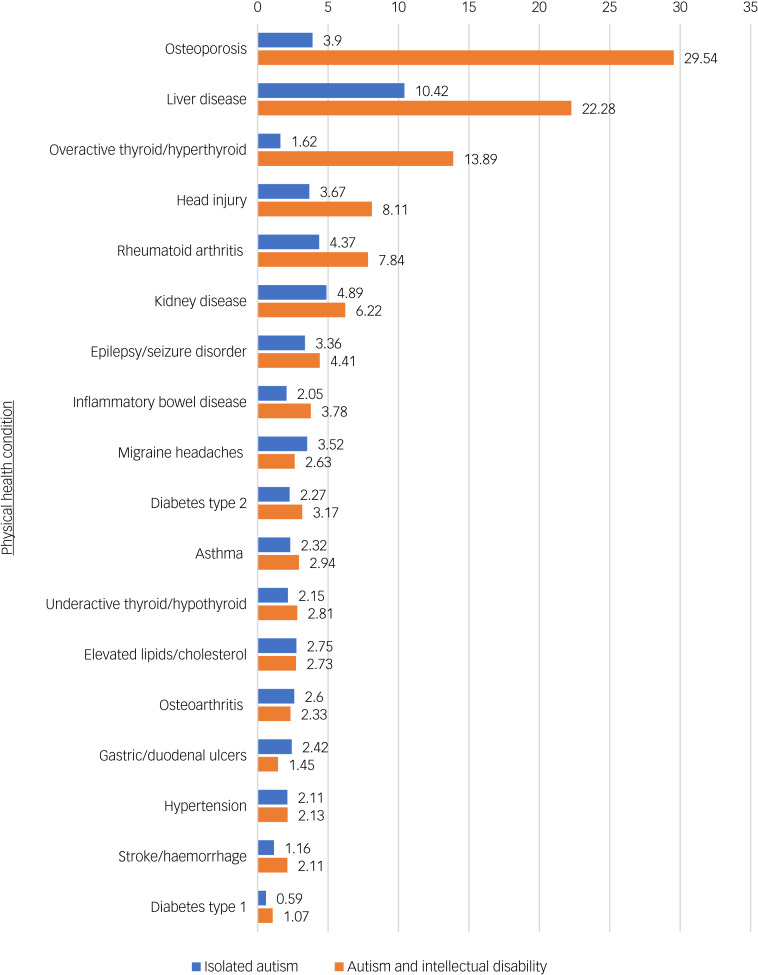
Graph showing the odds ratios from subanalysis for physical conditions in isolated autism or autism with intellectual disability, compared with the control population.

In the intellectual disability subanalysis logistic regression model, most conditions had similar odds ratios in both the isolated autism sample and the autism and intellectual disability sample (Table 3 and Fig. 1); however, fewer results reached significance in the intellectual disability subgroup. Several conditions demonstrated larger odds ratios in the autism and intellectual disability group (Fig. 1). These included head injury (odds ratio 8.11, 95% CI 2.51–26.26), liver disease (odds ratio 22.28, 95% CI 3.75–132.51), osteoporosis (odds ratio 29.54, 95% CI 6.20–140.64) and hyperthyroidism (odds ratio 13.89, 95% CI 2.36–81.99).

**Table 3:** Odds ratios from the subanalysis for isolated autism and autism with intellectual disability

Physical health condition	Isolated autism		Autism and intellectual disability	
	Adjusted odds ratio (95% CI)	*P*-value	Adjusted odds ratio (95% CI)	*P*-value
Asthma	2.32 (1.80–2.98)	<0.001*	2.94 (1.60–5.42)	<0.001*
COPD	7.47 (1.81–30.87)	0.005*	0.00 (0.00)	0.999
Diabetes type 1	0.59 (0.12–2.91)	0.516	1.07 (0.10–11.43)	0.956
Diabetes type 2	2.27 (1.24–4.15)	0.008*	3.17 (0.86–11.76)	0.084
Elevated lipids/cholesterol	2.75 (1.73–4.36)	<0.001*	2.73 (0.94–7.90)	0.065
Epilepsy/seizure disorder	3.36 (1.89–5.96)	<0.001*	4.41 (1.41–13.82)	0.011*
Gastric/duodenal ulcers	2.42 (1.20–4.88)	0.013*	1.45 (0.18–11.75)	0.729
Head injury	3.67 (2.19–6.17)	<0.001*	8.11 (2.51–26.26)	<0.001*
Heart disease	0.60 (0.17–2.04)	0.409	0.00 (0.00–0.00)	0.997
Hypertension	2.11 (1.44–3.11)	<0.001*	2.13 (0.81–5.60)	0.124
Kidney disease	4.89 (1.87–12.81)	0.001*	6.22 (0.69–55.62)	0.102
Liver disease	10.42 (3.48–31.17)	<0.001*	22.28 (3.75–132.51)	<0.001*
Migraine headaches	3.52 (2.70–4.58)	<0.001*	2.63 (1.33–5.23)	0.006*
Osteoarthritis	2.60 (1.51–4.50)	<0.001*	2.33 (0.50–10.90)	0.282
Osteoporosis	3.90 (1.41–10.81)	0.009*	29.54 (6.20–140.64)	<0.001*
Rheumatoid arthritis	4.37 (2.21–8.66)	<0.001*	7.84 (1.66–37.04)	0.009*
Stroke/haemorrhage	1.16 (0.36–3.74)	0.800	2.11 (0.23–19.85)	0.512
Overactive thyroid/hyperthyroid	1.62 (0.47–5.56)	0.444	13.89 (2.36–81.99)	0.004*
Underactive thyroid/hypothyroid	2.15 (1.26–3.66)	0.005*	2.81 (0.77–10.33)	0.120
Inflammatory bowel disease	2.05 (1.03–4.09)	0.042	3.78 (0.70–20.39)	0.122

COPD, chronic obstructive pulmonary disease.

* Indicates *P*-values that are significant after Benjamini–Hochberg correction (α 0.029).

## Discussion

In this study, we investigated the prevalence of physical health conditions in autism compared with a control sample without autism or mental health conditions. The results suggest that individuals with autism are at an increased risk of a range of physical health conditions compared with individuals without autism, across multiple organ systems, and the risk for some conditions is elevated in individuals with comorbid intellectual disability. This continues into older adulthood, with diagnoses such as osteoporosis and dementia being significantly more common in individuals with autism. Such findings are in keeping with results of previous studies, which also find increased odds of a range of physical health conditions in autism, and here we add to that literature with novel associations with previously unstudied physical health conditions.^6–9^

In accordance with existing research, our study found increased odds of epilepsy in autism, although the adjusted odds ratio of 3.44 was lower than reported in previous studies.^7,8,11,13^ This may be accounted for by an unusually high frequency of epilepsy in the control sample (1.8% compared with an estimated 0.4–1.0% in the general population^32^), likely resulting from the ascertainment and self-selection bias present in the wider NCMH database recruitment. It is difficult to compare to previous literature, as studies are heterogenous in their definition of epilepsy, ranging from strict international criteria to simply more than one seizure, and in this study, the questionnaire reported data on ‘epilepsy/seizure disorder’, which could be interpreted by participants to include non-epileptic seizure disorders. Despite this, our study and findings from existing research support the conclusion that epilepsy is significantly more common in autism. This study also recorded an increased risk of migraine headaches, which mirrors findings by Underwood et al^33^ in an earlier, smaller sample of this cohort and similar findings in other studies,^10,13,29^ although not all found a significant association.^7,11,12^ Taken in combination, the results are in keeping with Pan et al^13^ and Ward et al,^10^ who found increased presence of neurological disorders in autism.

Our findings strongly support increased odds of liver and kidney disease in individuals with autism, with a nearly 11-fold and five-fold increased risk, respectively; however, existing research on these diseases is sparse. Renal disease was found to have a slightly increased odds in autism in the study by Croen et al^7^ (adjusted odds ratio 1.26, 99% CI 1–1.59), whereas Liu et al found no increased risk,^9^ but studies are few. Hepatic disease was found to have a non-significant increased odds in adults with autism in the study by Croen et al (adjusted odds ratio 1.58, 99% CI 0.96–2.60),^7^ whereas Shedlock et al found that children with autism were more likely to have non-alcoholic fatty liver disease/steatohepatitis than controls (odds ratio 2.74, 95% CI 2.06–3.65).^20^ A recent study by Ward et al found increased odds of hepatic/renal disease in individuals with autism,^10^ whereas Schott et al found decreased odds of hepatic (odds ratio 0.87 99% CI 0.83–0.91) and renal (odds ratio 0.82, 99% CI 0.80–0.84) disorders.^12^ The current results suggest liver and kidney disease may be much more common in the autistic community, but further investigation is required to examine whether this replicates across the autistic population or could be accounted for by confounders in our sample.

This analysis also found a trend toward increased metabolic diseases in autism, with increased odds of type 2 diabetes, elevated lipids and hypertension. This echoes findings by Shedlock et al^20^ of increased prevalence of obesity, type 2 diabetes, hypertension, hyperlipidaemia and fatty liver disease in children with autism. Indeed, the findings of increased hypertension and hyperlipidaemia fit with most previous findings on these conditions,^7,8,11,12,14,20^ suggesting they are more common in autism, but it is difficult to make conclusions about type 2 diabetes, given the mixed findings in existing literature. This study lacked obesity data, but obesity rates may also be higher in autism,^9,12,20,34^ leading to questions of whether the increased prevalence of these conditions is driven by diet and weight, or if independent mechanisms are at play.

The lack of significant findings for type 1 diabetes contrasted other autoimmune conditions, where we found increased odds in the autistic cohort, including rheumatoid arthritis, ‘other autoimmune disease’ and hypothyroidism (which, although not exclusively autoimmune in aetiology, is often caused by autoimmune disease). Overall, we add to the picture that autoimmune diseases are more prevalent in autism,^7,23,24^ although specifics of individual autoimmune conditions are still unclear.

Findings of significantly increased odds of osteoarthritis, osteoporosis and nominally significant increased odds of memory loss/dementia were in keeping with Croen et al's findings on dementia,^7^ and Hand et al's and Liu et al's findings of an increased risk of a range of age-related health conditions, including osteoarthritis,^9^ osteoporosis and cognitive disorders.^8^ Some researchers theorise that there may be overlapping pathophysiology between autism and neurodegenerative conditions like dementia and Parkinson's disease, including genetic commonalities, defects in neurotransmitters common to both conditions and changes in beta-amyloid seen in autism that may predispose to Alzheimer's disease.^35^ This research is in its infancy and many of the proposed mechanisms are currently theoretical, but it may in time yield concrete evidence of shared predisposing mechanisms. Few studies on physical health consider the older autistic population, despite many conditions increasing in prevalence with age, or only occurring in older individuals. This study had a relatively small sample of people aged >50 years in the autism sample (91 individuals, 14.4% of the sample), but its findings provide support to the small number of existing studies and suggest this is an area requiring substantial further investigation, as greater numbers of older adults with autism are identified, potentially including longitudinal studies into older adulthood.

Several findings in this study were novel. The study found increased odds of gastric/duodenal ulcers (adjusted odds ratio 2.37, 95% CI 1.18–4.76), which, to the authors’ knowledge, has not been documented in existing literature. This finding may relate to autistic behaviours, and therefore the potential impact of diet and medications like non-steroidal anti-inflammatory drugs on this relationship would be an interesting area for future exploration. Head injury is also not specifically explored in any existing epidemiological literature, despite being associated with autism.^36^ This study found significantly increased odds of head injury in autism (adjusted odds ratio 3.84, 95% CI 2.30–6.39). Exploration of the nature of head injuries and the cause behind them (e.g. behavioural, neurological/motor deficits, self-injury) is warranted.

For some conditions, no significant association with autism was found. Breast cancer, cancer and heart disease did not show significant differences in odds ratios in our sample. Research on these conditions is mixed, with studies finding higher,^8,29^ lower^11,12^ and non-significant^7^ odds of cancer compared with controls, and studies finding higher odds^7–9,21^ or no difference^9,14,22^ in heart disease in autism. This study also found no significant difference in stroke between groups, despite findings of increased odds in three previous studies.^7–9^ Other conditions, including multiple sclerosis, Parkinson's disease and HIV, had low absolute prevalence in the data-set such that they are underpowered to detect any differences between groups, and we cannot comment on their prevalence in autism.

Although we could not do a direct comparison between individuals with and without intellectual disability, we observed that for osteoporosis, hyperthyroidism, liver disease and head injury, there are larger odds relative to the control group in the group with concurrent intellectual disability compared with individuals with autism without intellectual disability. This suggests that this population may be at increased risk of these conditions; however, confidence intervals for both groups overlapped. Several systematic reviews conclude that epilepsy prevalence in autism is higher in those with concurrent intellectual disability,^15,16^ and one study in people aged >65 years found increased odds of several conditions, including osteoporosis, epilepsy, gastrointestinal disorders, thyroid disorders and cognitive disorders.^37^ Another study, however, found the risk of most physical health conditions, compared with individuals without autism, was similar in adults with autism with and without intellectual disability.^9^ This is a relative new area of study, limiting the ability to draw conclusions, especially given the size of our sample; however, these early results suggest that concurrent intellectual disability could confer increased risk for certain physical conditions.

The mechanisms underpinning increased physical health problems in autism are likely multifactorial, involving shared aetiological factors, genetic influences and differences in behaviour and lifestyle. It is possible that barriers to accessing appropriate healthcare, such as communication difficulties, different symptom presentation and sensitivity to examinations or healthcare settings, may limit preventative care, leading to a greater development of chronic disease in this population. Poor diet and exercise, which can be a result of behavioural restriction, food hypersensitivities, social difficulties and comorbid motor/nervous conditions, may be another contributing mechanism. Weir et al^38^ found that individuals with autism were less likely to meet recommendations for diet and exercise on most measures, and more likely to be underweight or obese than controls. These may increase the risk of a variety of negative health outcomes, such as cardiovascular disease. For epilepsy, there are many studies that evidence areas of overlap between the two conditions. This includes findings of dysfunction in GABA signalling and circuity, common associated genes and abnormal grey-white matter volumes in both autism and epilepsy.^39^ A hypothesised biological mechanism underpinning both conditions may be one of excitation–inhibition imbalance in the neural circuitry, leading to hyperexcitability, with different genetic or neurodevelopmental changes found to be common to both epilepsy and autism converging to cause this imbalance.^39^ It is possible that other shared mechanisms may exist, but are yet to be determined.

### Limitations

This was an analysis of existing data, collected over several years, using iteratively updated questionnaires. Several physical health outcomes, including COPD, head injury, HIV, meningitis, inflammatory bowel disease and ‘other autoimmune disease’ were not present in the earliest versions of the NCMH questionnaire, meaning that these had more missing data (20.2–20.7%) compared with other variables (0.6–1.3%). Furthermore, missing data in these variables was overrepresented in autism (approximately 50% missing data, compared with approximately 12% in the control sample). Thus, the findings on these conditions are less reliable and must be taken with more caution.

Additional factors limited the generalisability and interpretation of our findings. Rates of physical health conditions in control individuals in the NCMH database may differ from the wider general population, as evidenced by the elevated rate of epilepsy in controls here. This may be caused by ascertainment bias through recruitment from healthcare environments such as NHS waiting rooms, and self-selection bias for individuals with health concerns. Second, some lifestyle factors that could affect physical health in autism, like body mass index and alcohol use, could not be controlled for. Meta-analytic evidence suggests that rates of obesity are significantly higher in autism,^34^ and thus obesity, which is a risk factor for a variety of conditions, could be a confounder of the relationship found between autism and physical health. Other variables, like smoking, had differing responses between questionnaire versions, with some versions quantifying smoking and others only asking about smoking as a binary yes or no. For consistency, this study recorded smoking as a binary yes/no, taken from ‘lifetime ever smoked’, and this may miss the full effect of smoking on physical health.

Furthermore, our control sample was defined by lack of mental health conditions, meaning it is not fully reflective of the general population and, despite knowledge that there is a high burden of co-occurring mental health conditions (e.g. attention-deficit hyperactivity disorder, anxiety disorders, depressive disorders) among the autistic community,^4^ our study could not control for the effects of these on physical health outcomes. In addition, clinical notes were not available to confirm diagnoses, so the study relies on self-report of clinician diagnosis, which reduces the reliability of our estimates.

Our autism group had a much lower mean age and only a limited number of individuals over 50 years of age, which may explain the higher rates of cancer, heart disease, hypertension and osteoarthritis in the control group found in the initial *χ*^2^ analysis. Although statistical modelling allowed us to control for the effects of age, the lack of older individuals with autism for comparison may mean we are missing the full picture around the occurrence of conditions that are more common with age.

Although the overall sample size in our study was not small, it was still small enough that rarer conditions with lower prevalences had too few cases for comparison, and our sample size was smaller than many comparable studies looking at a range of comorbidities.^6–9,11,12^ Autistic and control samples were not matched on variables such as age and gender, in an attempt to maximise sample size for testing. This introduced additional issues, as controlling for variables such as age and gender is not optimal, and likely introduce biases. Furthermore, the high percentage of females in the control group (65.8%), as well as the higher percentage of females and lower percentage of individuals with intellectual disability in the autism group than would typically be expected, does raise questions over the representativeness of our sample, and again point toward ascertainment, response and self-selection biases.^40^

Subanalysis was limited by the small sample of autistic individuals with intellectual disability. Our sample included only 86 in this group, with some conditions including fewer than 30 recorded responses. Several conditions had no cases or too few cases for meaningful analysis in this population. It is also likely that there is a selection bias away from individuals with intellectual disabilities in the wider NCMH sample, particularly those that are more severe, as several of the recruitment methods and the use of written questionnaires may better suit those without intellectual disability. To combat this, the NCMH has been undertaking targeted recruitment within the intellectual disability community by using appropriately adapted measures. Ultimately, concerns around size and representativeness of our sample for subanalysis limits any conclusions we can make, and emphasises that the experiences of individuals with intellectual disability are underresearched and underrepresented in the literature.

### Clinical implications and future directions

This study draws attention to the increased physical health burden experienced by the autistic community, and adds to a growing field of research in this area with novel associations. This increased health burden is likely to have wide-ranging effects on quality of life and mental health, and may contribute to a risk of premature mortality in autism.^41^ It is vital that research is carried out in all demographics of the autistic community, as some groups, such as older individuals (>50 years) or those with concurrent intellectual disability, are harder to sample and at risk of involuntary exclusion while also being more at risk. Further investigations are required to begin to understand the mechanisms behind the higher rates of physical health conditions observed in individuals with autism, alongside any mediating factors, with the aim of developing intervention and prevention strategies. Furthermore, there may be a role for enhanced health screening in individuals with autism, and future research should identify the value and focus of such screening. It is hoped that it will increase healthcare professionals’ awareness of physical health in autism, and encourage clinicians to have a lower threshold for considering physical illness when individuals present with changes in behaviour, as well as physical signs.

## Supporting information

Hunt et al. supplementary material Hunt et al. supplementary material

## Data Availability

The data that support the findings of this study are available on request from the corresponding author, J.F.G.U. The data are not publicly available due to NCMH ethics and data management requirements.
