# Risk of physical health comorbidities in autistic adults: clinical nested cross-sectional study – CORRIGENDUM

**DOI:** 10.1192/bjo.2025.57

**Published:** 2025-06-27

**Authors:** Megan Hunt, Jack F. G. Underwood, Leon Hubbard, Jeremy Hall

In the above published article, the authors’ intentional use of identity-first language was amended to person-first language during production.
