# Supplementary material for: Risk of physical health comorbidities in autistic adults: clinical nested cross-sectional study
Source: BJPsych Open. 2024 Oct 23;10(6):e182. doi: 10.1192/bjo.2024.777 (PMC11698207; doi:10.1192/bjo.2024.777)
Supplement: Hunt et al. supplementary material [file S2056472424007774sup001.docx]

Supplementary Materials

# Background

The National Centre for Mental Health (NCMH) is a Welsh Government funded Research Centre established in 2011, which has since enrolled >25,000 participants^1^. NCMH recruits both child and adult participants via NHS services and from the general population, with a specific focus on neurodevelopmental and psychiatric disorders^2^. Phenotypic information is obtained (i.e. information about the various manifestations of mental ill health including the nature, timing and persistence of clinical symptoms and signs as well as other psychological and biological variables), as well as biological samples, which can then be linked to routine clinical National Health Service (NHS) data^2^. The long-term aim of NCMH is to improve diagnosis and to develop and evaluate ways of preventing the onset, and improving the treatment and management of mental disorders through a better understanding of their biological, psychological and social causes^2^. NCMH is operated as a partnership by Cardiff University, Swansea University and Bangor University in collaboration with NHS Health Boards across Wales and NHS Trusts in England^2^.

NCMH Included conditions:

NCMH recruits Individuals with a history of developmental/mental disorder and/or a learning disability, with relatives of these individuals also invited to take part. To allow for comparison, control individuals without a history of developmental/mental disorder and/or learning disability are also invited to participate^2^.

NCMH Inclusion Criteria

Inclusion Criteria 1: Adult participants aged 18 or over with no upper age limit.

Inclusion Criteria 2: Child/teenage participants between the ages of 4 and 17.

NCMH Exclusion Criteria

Exclusion Criteria 1: Inability to understand spoken and/or written English and/or Welsh.

Exclusion Criteria 2: Inability to provide valid informed consent (for example, should the individual lack capacity to consent and there is no personal/nominated consultee available).

Individuals with blood-borne infection or past intravenous drug use without a recent negative blood screen are excluded from giving blood or skin samples.

NCMH Participant Recruitment & Identification

An aim of NCMH is to provide as many people as possible with the opportunity to participate in the research^2^. A range of approaches is used to bring the opportunity to the attention of potential participants. Approaches include^2^:

- Working with NHS Health Boards in Wales and NHS Trusts in England to establish and implement ways of offering potential participants in Primary and Secondary Inpatient and Outpatient Services the opportunity to take part.
- Working with NHS Child and Adolescent Mental Health Service (CAMHS) clinics across Wales and England to identify participants.
- Liaising with voluntary organisations and determining appropriate ways of bringing the research to the attention of individuals with mental disorders.
- Attending events and conferences to raise awareness of NCMH and to provide an opportunity for individuals to hear more about the research.
- Inviting individuals already enrolled in previous/ongoing research studies (for example, studies within Cardiff University and external studies such as Health Wise Wales (www.healthwisewales.gov.wales)), where participants have indicated their willingness to be approached about future studies.
- Using local, national and social media to inform the public/potential volunteers about the project.
- Using the NCMH website to inform the public/potential volunteers about the research (see www.ncmh.info).
- Inviting students at various Universities to take part.

# NCMH Brief Assessment

Physical Health Conditions

The NCMH Brief Assessments has been iteratively updated over the lifetime of the centre. The earliest version of the brief assessment included 22 physical health conditions, with this list gradually being expanded to 28. Where individuals had not been asked about a specific condition this was coded as NA, and excluded from the analysis. Versions 1.0, 1.1 and 1.2 of the NCMH questionnaire include 3 options for physical health conditions – ‘yes’, ‘no’ and ‘not sure’. Given that ‘not sure’ is ambiguous and does not indicate the definite presence or absence of any condition, such responses were excluded from analysis. The most recent online survey, the NCMH Student Cohort 2020, collapsed physical health questions into broader groups of organ systems (e.g. cardiovascular disease, respiratory disease); given the lack of specificity over which condition each individual has, these were also excluded. No statistical analyses were run for breast cancer, HIV and Parkinson’s disease as there were no cases in the autistic sample. After manual inspection, further physical health variables were removed prior to statistical analysis. The ‘structural heart problems’ variable was excluded due to there being only 13 responses and 99.6% missing data for this variable, along with the ‘other’ variable on account of its ambiguity, both in terms of what diagnosis it may represent and the number of diagnoses it may represent.

Age & Gender

Where age was computed from date of birth, this was rounded to integers. Gender data were reduced for analysis by combining the transgender female, transgender male, gender variant/non-conforming and other responses into one variable of transgender/gender non-conforming, as many of the individual gender identities had too few individuals in the sample to be used as individual covariates in the regression analysis.

Smoking, antipsychotic and mood stabilisers as co-variates

Smoking was measured as a binary lifetime ever variable and selected due to the well-established effects of chronic smoking on physical health outcomes^3^. Antipsychotic and mood stabiliser usage were also recorded as binary lifetime ever variables and selected to account for the common adverse physical side effects associated with these drug classes. These include, but aren’t limited to, metabolic effects, increased risk of diabetes mellitus, cardiovascular disease, stroke and agranulocytosis for antipsychotics^4^ and effects on thyroid function, liver function, kidney function and more modest effects on weight gain and risk of diabetes for mood stabilisers^4,5^. Both classes of medications could impact an individual’s physical health, and were therefore incorporated as co-variates to control for their potential use and physical effects in the autism cohort when compared to a control group without mental health disorders. We did consider the possibility of collider bias when investigating epilepsy, given that several mood stabilisers are also anticonvulsant medications and therefore may be seen at an increased rate in individuals with epilepsy, independent of autism. We therefore removed mood stabilisers as a co-variate in a post-hoc analysis to test for potential effects.

Post-hoc analysis was carried out for the variable epilepsy to check for possible collider bias when controlling for mood stabilisers. Original analysis, where mood stabilisers were included as a control, produced an OR of 3.44 (95% CI 1.95 – 6.04), while post-hoc analysis where mood stabilisers were not included as a control produced an OR of 3.30 (95% CI 1.88 – 5.76). These results showed minimal change in OR and confidence interval upon removal of mood stabilisers as a control. Furthermore, if collider bias were present, we would expect the OR to be lower when mood stabilisers are controlled for, which was not the case. Given these results, we decided to use the same model for epilepsy as for other conditions.

**References**

1. National Centre for Mental Health. NCMH Annual Report 2022-23. 2023. Available from: https://healthandcareresearchwales.org/sites/default/files/2024-02/NCMH-annual-report-2022-23-English-WEB.pdf

2. National Centre for Mental Health. NCMH_Protocol (v1.5). 2018.

3. NIDA. What are the physical health consequences of tobacco use? 2021 Apr.

4. Correll CU, Detraux J, De Lepeleire J, De Hert M. Effects of antipsychotics, antidepressants and mood stabilizers on risk for physical diseases in people with schizophrenia, depression and bipolar disorder. World Psychiatry. 2015;14(2):119–36.

5. Grootens KP, Meijer A, Hartong EG, Doornbos B, Bakker PR, Hadithy AA, et al. Weight changes associated with antiepileptic mood stabilizers in the treatment of bipolar disorder. Eur J Clin Pharmacol. 2018;74(11):1485–9.
